# Safety Aspects of the Use of Isolated Piperine Ingested as a Bolus

**DOI:** 10.3390/foods10092121

**Published:** 2021-09-08

**Authors:** Rainer Ziegenhagen, Katharina Heimberg, Alfonso Lampen, Karen Ildico Hirsch-Ernst

**Affiliations:** German Federal Institute for Risk Assessment (BfR), Max-Dohrn-Str. 8-10, 10589 Berlin, Germany; rainer.ziegenhagen@bfr.bund.de (R.Z.); Katharina.Heimberg@bfr.bund.de (K.H.); Alfonso.Lampen@bfr.bund.de (A.L.)

**Keywords:** piperine, food safety, drug interaction, reproductive toxicity, bolus administration

## Abstract

Piperine is a natural ingredient of *Piper nigrum* (black pepper) and some other *Piper* species. Compared to the use of pepper for food seasoning, piperine is used in food supplements in an isolated, concentrated form and ingested as a bolus. The present review focuses on the assessment of the possible critical health effects regarding the use of isolated piperine as a single ingredient in food supplements. In human and animal studies with single or short-term bolus application of isolated piperine, interactions with several drugs, in most cases resulting in increased drug bioavailability, were observed. Depending on the drug and extent of the interaction, such interactions may carry the risk of unintended deleteriously increased or adverse drug effects. Animal studies with higher daily piperine bolus doses than in human interaction studies provide indications of disturbance of spermatogenesis and of maternal reproductive and embryotoxic effects. Although the available human studies rarely reported effects that were regarded as being adverse, their suitability for detailed risk assessment is limited due to an insufficient focus on safety parameters apart from drug interactions, as well as due to the lack of investigation of the potentially adverse effects observed in animal studies and/or combined administration of piperine with other substances. Taken together, it appears advisable to consider the potential health risks related to intake of isolated piperine in bolus form, e.g., when using certain food supplements.

## 1. Introduction

The alkaloid piperine ((E,E)-piperine; IUPAC-name: (2E,4E)-5-(2H-1,3-benzodioxol-5-yl)-1-(piperidin-1-yl)penta-2,4-dien-1-one; CAS-No.: 94-62-2; FEMA-No: 2909; molecular formula: C_17_H_19_NO_3_; molecular weight: 285.34 g/mol) is a natural ingredient of *Piper nigrum, Piper longum* and some other *Piper* species, as well as of *Aframomum melegueta* K. Schum. (Grains of Paradise) [1,2,3,4,5,6,7]. The alkaloid is the main compound imparting the pungent flavour to fruits of *Piper nigrum* and *Piper longum*. *Piper nigrum* fruits are used to produce black, white and green pepper. Black peppercorns are produced from whole, dried, full-grown, not yet fully ripe fruits, while white peppercorns are produced from dried, ripe fruits after removal of the outer layer [8,9]. Green peppercorns are obtained from unripe fruits subjected to processing methods by which the green colour is maintained.

The occurrence of piperine in the European/Western diet primarily results from use of pepper for food seasoning, but also from the use of the substance in isolated form for spicing/flavouring purposes, e.g., in beverages and spirits [2]. The substance can occur in four stereoisomeric forms: (E,E)-piperine (= piperine), (Z,E)-piperine (= isopiperine), (E,Z)-piperine (= isochavicine) and (Z,Z)-piperine (= chavicine). In black and white pepper, (E,E)-piperine constitutes by far the main and most pungent isomer. The other three isomers seem to be formed primarily via light-induced or enzymatic isomerization [10].

Primarily in in vitro and in animal studies, as well as in some human studies, piperine has been shown to be a biologically versatile compound that can interact with a variety of chemically and functionally diverse biomolecular targets, such as enzymes, membrane transporters, receptors or other biomolecules. For example, piperine may provide protection against forms of oxidative damage and improve the activities of compromised anti-oxidative defence mechanisms (e.g., related to superoxide dismutase or catalase), but depending on the study settings, may also decrease the anti-oxidative defence mechanisms and among others, piperine further displays the potential to influence the activity of drug-metabolizing enzymes, including enzymes involved in phase I (cytochrome P-450-enzymes) and phase II metabolism (e.g., UDP-glucuronosyltransferases), to interact with cellular drug transporters (e.g., P-glycoprotein) or to modulate the cellular targets (monoamine oxidase) associated with neurodegenerative diseases [1,11,12,13,14,15,16,17,18].

Currently, attention is largely focused on the potential of piperine to influence the bioavailability of certain drugs via interaction with drug-metabolizing enzymes and/or inhibition of drug transporters or efflux pumps, thereby in many cases increasing the drug bioavailability and efficacy. In addition, inclusion of piperine into drug-loaded nanoparticles or lipospheres is being investigated as a means of increasing the effectiveness of advanced drug delivery systems [19,20].

In food supplements, piperine (primarily in the form of highly piperine-enriched pepper extracts, frequently with a piperine content in the range of ≥ 95%) is often used and promoted, among others, as a bio-enhancer to increase the bioavailability of other ingredients contained in these food supplements. Based on the use of piperine in various food supplements, its multi-facetted biological activities, and on the differences regarding the pattern of piperine intake when comparing the use of food supplements to the use of pepper for food seasoning, a closer look into the safety aspects of the use of piperine as an ingredient of food supplements appears to be warranted. 

This review focuses on the possible critical health effects regarding the use of isolated piperine as a single ingredient in food supplements (i.e., without the addition of other bioactive substances). The use of isolated piperine as a flavouring agent is outside the scope of this review. In the context of the present review, the focus was laid on adult persons; thus, children and adolescents were not considered. To this end, a literature search was performed in the scientific databases Pubmed and Embase, with the last update performed in February 2021. To initially retrieve a broad spectrum of references, the search term “piperine” was used, without combination with other search terms. To further identify the relevant scientific publications that are within the scope of the present review, the abstracts of the retrieved references were screened to facilitate the selection of a subset of publications that were subsequently subjected to further scrutiny of the full texts. In addition, reference lists of the identified relevant publications as well as websites of acknowledged scientific bodies or national authorities were checked.

## 2. Occurrence and Exposure

### 2.1. Occurrence

*Piper nigrum* is the main source of piperine in European/Western cuisine. Other potential sources are foods flavoured with piperine in isolated form or foods flavoured with other *Piper* species (e.g., *Piper longum* and *Piper retrofractum* Vahl)or with the spice “grains of paradise” (*Aframomum melegueta*) [2]. Regarding the piperine content of black pepper, ranges of 2–7% [21], 2–9% [13] or 4–6% with contents up to 10% [2] have been reported. For *Piper longum*, piperine contents of 1.2–5% [13,22,23,24], and for *Piper retrofractum* Vahl contents of 3.1–4.5% have been indicated [13,23]. In an investigation of four commercial brands of pure ground black pepper with high piperine contents (10–11%), E,E-piperine was the most abundant (≥99% of detected piperine isomers) and Z,Z-piperine (= chavicine) the least abundant (≤0.07%) piperine isomere [10]. 

During storage of ground black, white and green pepper at 4 °C for 6 months, a decrease in piperine content of about 12–30% was observed [25]. Different findings were made regarding heat treatment of pepper, ranging from mild piperine losses of 4–12.5% during cooking in an open pan (30 min) or pressure cooking (20 min) [26] to losses of approximately 28% during cooking (20 min) or about 34% during pressure cooking (10 min) [27]. 

### 2.2. Exposure

In 2007, by extrapolation from limited consumption data, the Australian Therapeutic Goods Administration (TGA) estimated that the piperine intake in New Zealand was about 25 mg per person per day, in the USA approximately 60 mg and in India approximately 120 mg per person per day [28]. According to another source, which was based on annual US import data of black pepper, with an estimated average per capita intake of approximately 0.7 g pepper/day, a corresponding per capita piperine intake of 14–54 mg/day was calculated for the US population [29].

In an exposure estimation performed by the German Federal Institute for Risk Assessment (BfR) in 2018, which was based on food consumption data from the National Consumption Survey II of the Max Rubner-Institute (2008) [30], including approximately 20,000 individuals, a mean per capita pepper intake of the male German population (14–80 years) of 0.6 g/day was estimated, with an estimated per capita intake at the 95th intake percentile of 1.6 g pepper/day. Assuming an average piperine content in pepper of 4–6%, this would correspond to an estimated mean per capita intake by the male population of 24–36 mg piperine/day and an estimated intake at the 95th percentile of 64–96 mg piperine/day. Regarding this estimation, it is noted on the one hand that information on the consumption of herbs/spices is generally subject to greater uncertainty as their consumption is often not documented, and an underestimation of the amount consumed can therefore be assumed. On the other hand, it should be borne in mind that this intake estimation does not take into account possible piperine losses caused by storage or food preparation. 

In India, a consumption survey conducted from December 2006 to July 2008 in three regions recorded the median monthly per capita intakes of black pepper of 3–18.5 g (0.1–0.62 g/day) and in the 90th percentile of 16.7–41.7 g (0.56–1.39 g/day) [31]

Taken together, the available estimations of daily piperine intake resulting from the use of pepper in food preparation are afflicted with considerable scientific uncertainty. It should also be kept in mind that when using pepper for food seasoning, the piperine intake occurs in conjunction with all other pepper constituents and with different degrees of comminution of the peppercorn, potentially bringing about matrix effects influencing the bioavailability or pharmacodynamic effects of piperine ingested in this way, which may differ from the intake of piperine as an isolated substance.

In its assessment of isolated piperine and several aliphatic and arylalkyl amines and amides as flavouring agents, the European Food Safety Authority EFSA (2015) reported an estimated European per capita intake of 6.2 µg piperine/day for the use of isolated piperine as a flavouring substance based on the EU Maximised Survey-derived Daily Intake (MSDI) method (see also below). However, EFSA noted in this assessment that the use levels were needed for some of the abovementioned flavouring substances, including piperine, to calculate the Modified Theoretical Added Maximum Daily Intakes (mTAMDIs) in order to identify those flavouring substances that required a more refined exposure assessment and to finalise the evaluation [32].

In food supplements, piperine is usually used in combination with other ingredients to increase their bioavailability, and commonly its addition occurs via highly piperine-enriched black pepper extracts (piperine content frequently in the range of ≥ 95%). Therefore, black pepper extract is often mentioned on the ingredient list of food supplements and the piperine content is only indicated in second place. The piperine content of food supplements is frequently in the range of 5–30 mg per daily dose, with single products reaching dosages of 40 or up to about 50–100 mg per daily dose, but the market may be subject to change.

The piperine content of the highly piperine-enriched black pepper extracts (frequently in the range of ≥95%) is very similar to the piperine content of chemically defined piperine used as an isolated flavouring substance (piperine content ≥97% [33]) or to the piperine content of the substance used in scientific investigations, for which it was procured as a chemical from chemical companies (usually ≥97%).

## 3. Kinetics and Metabolism

In animal studies conducted by Bhat and Chandrasekhara [34,35] and Suresh and Srinivasan [36], with rats receiving an oral dose of 170 mg piperine/kg body weight (bw), only about 3–4% of the dose was detected in faeces in unchanged form over a period of 4 or 5 days, respectively, and it was concluded that 96–97% of the administered piperine dosage was absorbed [34,36]. In an accompanying investigation with everted sacs of rat intestines, only piperine was detected in serosal fluid and intestinal tissue, which led to the conclusion that piperine did not undergo any metabolic change during absorption [34]. However, in both the abovementioned animal studies, only small portions of the administered oral dose could be detected in serum and investigated tissues. In the more recent study of Suresh and Srinivasan (2010), maximum levels were reached 6 h after oral administration of a piperine dose of 170 mg/kg bw, with approximately 38.8 µmol piperine/L in serum and 0.39% of the administered piperine dose in liver, 0.37% in kidney and about 9.7% in the flushed intestine [36]. In both studies, no piperine was detectable in urine [34,36], but Bhat and Chandrasekhara detected piperine metabolites, i.e., piperonylic acid, piperonyl alcohol, piperonal and vanillic acid, and their conjugates, in urine, which in their free forms represented about 15.5% of the administered dose (measured within 96 h after piperine administration) [35]. The latter authors assumed that most of the administered piperine was absorbed and that it was not transformed during intestinal absorption but was probably later metabolized rapidly by other tissues [34]. 

In a more recent study in rats, the bioavailability of an oral dose of 3.5 mg piperine/kg bw was calculated to be about 25% by comparing plasma AUC values following oral and i.v. administration [37]. Regarding piperine metabolites, Gao et al. (2017) identified 12 metabolites in rat plasma, bile, urine and faeces, with 10 piperine metabolites occurring both in plasma and urine. The metabolites were grouped into metabolites resulting from methylenedioxycyclic ring-opening, from methylenedioxycyclic ring-oxidation and from piperidine ring-cleavage [38]. Shang et al. (2017) even detected and tentatively characterized 148 piperine metabolites in rat plasma, urine and faeces after oral administration of 250 mg piperine/kg bw. Piperine mainly underwent hydrogenation, dehydrogenation, hydroxylation, glucuronide conjugation, sulphate conjugation, ring cleavage and their composite reactions. However, information on plasma or urine levels of the detected piperine metabolites is not available from this study [39]. In laying hens receiving piperine–enriched feed (80 mg/kg feed), significant proportions of piperine isomers were observed in egg yolks (3.0 µg piperine, 0.7 µg chavicine, 2.9 µg isopiperine and 5.3 µg isochavicine per g egg yolk), indicating that piperine metabolism can also comprise substance isomerization [40].

Information on piperine serum or plasma levels observed in rats after oral administration is not uniform. With oral doses of 3.5, 20, 35 or 250 mg piperine/kg bw, corresponding plasma C_max_ values of approximately 0.45, 3.4, 5.4–6.0 or 12.7 µmol piperine/L were observed in different studies [37,41,42,43]. However, other studies observed higher C_max_ values with approximately 9.9 µmol/L after an oral dose of 20 mg piperine/kg bw [44] or levels of approximately 28–39 µmol/L after a dose of 170 mg piperine/kg bw [34,36]. Plasma protein binding was about 98% in rats receiving an oral dose of 35 mg piperine/kg bw [42]. Furthermore, piperine was shown to efficiently penetrate and homogeneously distribute into the brain of rats after oral piperine doses (35 mg/kg bw), leading to comparable AUC_(0-∞)_-values in brain and plasma with a brain–plasma AUC ratio of 0.95. However, based on the AUC_(0-∞)_ values, the piperine level in cerebrospinal fluid was around 50 times lower than in brain or plasma [42]. 

In humans, information on kinetics and metabolism of oral piperine doses are sparse. In an investigation with two individuals receiving a single oral dose of 50 mg piperine (approximately 0.71–0.83 mg/kg bw, assuming a body weight of 60–70 kg), the plasma peak concentrations reached 2.7–3.3 µmol/L (T_max_ = 1–3 h) [45]. 

In human urine, the piperine metabolites 5-(3-4-dihydroxphenyl)valeric acid piperidide (which was excreted as sulphate) and its derivate hydroxylated in position 4 of the piperidine ring, 5-(3-4-dihydroxphenyl)valeric acid-4-hydroxypiperidide, were observed one or two days after oral administration of piperine (25 mg) or a high dose of pepper, respectively. Interestingly, these two urine metabolites could not be detected in 2 out of 14 investigated individuals who instead excreted 5-(3-4-dihydroxphenyl)-2-4-pentadienoic acid piperidide, providing first indications for individual differences in human piperine metabolism [46]. In rat urine, all three metabolites could be detected [38]. In an in vitro study comparing the hepatic piperine metabolism in mouse, rat, dog and human hepatocytes, the predominant metabolic pathways included formation of a catechol derivate for all species; however, the metabolic pathways displayed species-specific differences in terms of types and quantities of metabolites [47]. 

## 4. Safety Aspects

### 4.1. Information Based on Evaluations by Scientific Bodies and National Authorities 

The European Food Safety Authority (EFSA) has evaluated the use of piperine as a flavouring substance. In its evaluations, EFSA (2008; 2011; 2015) disagreed with a No Observed Effect Level (NOEL) of 20 mg piperine/kg bw/day that had previously been identified by the Joint FAO/WHO Expert Committee on Food Additives (JECFA) (2006), due to the shortcomings of the underlying animal study (lack of histopathology, study duration) and in 2015 identified a No Observed Adverse Effect Level (NOAEL) of 5 mg piperine/kg bw/day, based on a newly available 90-day rat feeding study performed according to OECD guideline 408 (endpoint: dose-dependent increase in cholesterol level in male animals) [29,32,48,49,50] (see (2) in Section 4.2.2). In its final conclusion, EFSA (2015) agreed with the JECFA (2006) conclusion “no safety concern at estimated levels of intake as flavouring substance” based on the MSDI approach (estimated European per capita intake by the MSDI approach: 6.2 µg piperine/day) [32,50]. Currently, isolated piperine is approved as a flavouring agent in the European Union with no restrictions on use or maximum levels set in regulation (EC) No. 1334/2008. However, its use level may be self-limiting due to the pungent taste of piperine.

In 2007, the Australian Complementary Medicines Evaluation Committee (CMEC) evaluated the use of piperine as a component in herbal preparations for use in listed medicines. Due to the possible effects on the bioavailability of medicinal products (leading to increased bioavailability in most cases, see Section 4.2.4) and the risk of inadvertent interactions with medicinal products, the committee recommended a maximum daily dose limit of 10 mg/day for piperine (based on a person’s body weight of 50 kg) when present as a component in herbal preparations for use in listed medicines [28].

The Canadian authority Health Canada (2019) has elaborated a monograph on the use of *Piper nigrum* (black pepper) as an ingredient in *Natural Health Products,* which also includes piperine isolated from the fruits of *Piper nigrum*. For adults (≥18 years), a daily dose of 250–420 mg for the unextracted powder of *Piper nigrum* fruits and a daily maximum dose of 14 mg for the use of piperine as an isolated substance in these products were established. For these products, a label statement is required that persons taking other medicines or natural health products should consult a healthcare practitioner/provider/professional or physician before use, as black pepper/piperine may alter their effectiveness. The same applies to pregnant or breastfeeding women. The monograph does not list any contraindications or known adverse reactions [51].

None of the evaluations described above mentioned the paternal reproductive toxicological effects observed in some animal studies (see (3) in Section 4.2.2).

In 2016, on request of the Norwegian Food Safety Authority, the Norwegian Scientific Committee for Food Safety (VKM) carried out a risk assessment of a daily dose of 1.5 mg piperine in food supplements. The panel applied the Margin of Exposure (MOE) approach in its assessment and used the NOAEL of 5 mg/kg bw/day identified by EFSA (2015), which was based on an animal study (endpoint: dose-dependent increase in cholesterol level in male animals) as the starting point for the MOE calculation. As the margin of exposure for all age groups considered was greater than 100, the panel concluded that this intake was unlikely to produce adverse effects in individuals aged 10 years or older [2].

### 4.2. Potential Hazards

#### 4.2.1. Genotoxicity

In its assessment of piperine as a flavouring agent, JECFA (2006) concluded regarding genotoxicity that piperine belongs to a group of aliphatic and aromatic amine and amide derivates for which negative results were reported in bacterial assays for reverse mutation and that piperine consistently gave negative results in a variety of in vivo studies [50,52,53,54]. EFSA agreed in its assessment with JECFA that the available studies on genotoxicity did not preclude the evaluation of piperine (and some other aliphatic and arylalkyl amines and amides) as a flavouring agent [32,48,49]. 

In a more recent study, piperine displayed negative results in an in vitro micronucleus test with Chinese hamster ovary cells in the presence or absence of metabolic activation and caused no increase in the numbers of micronucleated polychromatic erythrocytes in an in vivo micronucleus test in mice with all the tested doses (highest tested dose 574 mg/kg bw), leading to the conclusion that in this study piperine was not genotoxic [55].

#### 4.2.2. Animal Studies

(1) Acute and Subacute Toxicity

Piyachaturawat et al. investigated the acute toxicity of piperine (dissolved in equal volumes of DMSO and 95% ethanol) following a single administration via intragastric (i.g.) gavage to mice and rats [56]. For adult male mice, the resulting calculated LD_50_ value was 330 mg/kg bw, compared to an LD_50_ of 15.1 mg/kg bw based on i.v. administration. In the same study, an LD_50_ value of 514 mg/kg bw was derived for single i.g. administration of piperine to adult female rats, while a higher LD_50_ value was calculated for young female weanling rats (LD_50_ > 585 mg/kg bw). Animals receiving lethal doses experienced convulsion and died of respiratory paralysis [56]. 

In another study with mice, all animals survived oral doses of 143 to 574 mg/kg/day given on two consecutive days, but displayed lethargy ranging from a slight degree in the low-dose group to a severe one in the high-dose group [55]. 

In a subacute oral toxicity study with female rats receiving 0, 100, 250, 350 or 500 mg piperine/kg bw/day for seven days, the body weight gain of animals receiving 100 mg/kg bw was comparable to that of the control group. Daily doses of 250 mg/kg bw led to reduced body weight gain and caused haemorrhage in the stomach of 3 out of 8 animals, whereas the higher doses of 350 and 500 mg/kg bw caused the death of 2 and 5 out of 8 animals, respectively. Animals receiving 500 mg piperine/kg bw/day displayed histopathological changes of different types and degrees in the stomach, urinary bladder, adrenal glands and small intestine. In addition, luteal cells in the central portion of the corpora lutea were degenerated in this dosage group [56].

(2) Subchronic Toxicity Studies

In a study with groups of young male rats that received 100 mg piperine/kg feed for 56 days or 110, 220 or 440 mg pepper oleoresin/kg feed (equivalent to 50–200 mg piperine/kg feed), or 2 g pepper/kg feed, no adverse effects on growth, food efficacy, organ weights, blood count and investigated clinical chemistry parameters were observed compared to the control group [50,57]. In its evaluation of piperine as a flavouring agent, JECFA (2006) based its NOEL for piperine of 20 mg/kg bw/day on this animal study, but EFSA (2008) considered this study inadequate for NOAEL identification due to study-design limitations [48,50]. It should be mentioned that this animal study reported increased haemoglobin values for the piperine group (242 g/L) compared to the control and to the pepper/pepper oleoresin groups (~140 g/L); however, the red blood cell count was similar to the other groups, making a typing error highly possible [57].

The subchronic toxicity study (90-day study) in rats that was finalised in 2013 and used by EFSA (2015) [32] for the assessment of piperine as a flavouring agent has been published meanwhile (Bastaki et al. (2018) [29]). In this study, 0, 5, 15 or 50 mg piperine/kg bw/day were administered via feed for 90 days. According to the EFSA assessment, the reduced weight gain observed in the highest male dose group was due to reduced feed intake (possibly related to food palatability). There were no mortalities, no gross and microscopic changes nor clinical pathology or organ weight changes attributable to piperine. Some statistically significant changes in haematology, coagulation or clinical chemistry parameters were considered by EFSA as not dose-dependent, small in magnitude and within the range of historical values. However, statistically significant dose-dependent increases in cholesterol levels were observed in male animals receiving 15 and 50 mg piperine/kg bw/day (approximately by 30 and 55%, respectively), which was used by EFSA for NOAEL identification. 

According to EFSA, reduced relative epididymides weights were observed in male animals administered 5 and 50 mg/kg bw/day, respectively, but these changes were considered small and not dose-dependent and therefore of limited toxicological relevance [32]. Regarding this finding, it is noted on the one hand that reduced relative (to-brain weight) epididymis weights (17–23%) were observed in all three piperine dosage groups without displaying dose dependence, with reductions being statistically significant only in the 5 and 50 mg/kg bw dosage groups. On the other hand, it must be mentioned that these changes were without histopathological findings. From this study, EFSA identified a NOAEL of 5 mg piperine/kg bw/day due to the dose-dependent elevated cholesterol plasma levels in male animals at the mid and high dose (15 and 50 mg/kg bw/day) [32]. 

Contrary to the EFSA (2015) assessment, Bastaki et al. (2018) ascribed no toxicological relevance to the observed increases in cholesterol levels described above because, in their opinion, the cholesterol levels were within the historical control range for male animals and because of corroborating evidence from other studies showing an absence of a cholesterol increase. Rather, they identified a NOAEL of 50 mg piperine/kg bw/day (the highest dose tested) from this study [29].

(3) Paternal Reproductive Toxicological Effects

In 1999, as a consequence of administration of 0, 5 or 10 mg piperine/kg bw/day (suspended in 0.9% saline) via a gastric catheter to young adult male rats for 30 days (n = 10 per group), Malini et al. [58,59] reported in the high-dose group statistically significantly (*p* < 0.05) reduced sperm concentrations in caput and cauda epididymides, statistically significantly reduced relative weight of testis (relative to body weight) and reduced absolute weight of the cauda epididymides, vas deferens, seminal vesicle and ventral prostate. The relative organ weights were also reduced, according to the authors’ own calculations based on the mean body and organ weights stated in one of the two publications by Malini et al. [59]; however, no information on statistical significance is available for these calculations. Furthermore, histopathological changes in the testis, increased serum gonadotropins (FSH, LH) and reduced the intra-testicular testosterone concentration, as well as reduced the testicular lipid content, changes in the testicular lipid profile and reduced activity of some testicular lipogenic enzymes were observed (for details, see Table 1). Histopathological changes in the testes were also observed at 5 mg piperine/kg bw/day, but to a lesser extent than in the high-dose group. Within this low-dose group, the other parameters mentioned above showed changes in the same direction as seen in the high-dose group, but these changes were also of smaller magnitude and, in the majority of cases, no longer statistically significant. The reduced testicular weight was attributed to disturbed spermatogenesis and the reduced total lipid content of the testes caused by piperine administration [58,59]. 

In another study [60], D’Cruz and co-workers administered 0, 1, 10 and 100 mg piperine/kg bw/day dissolved in a vehicle (10% DMSO in ethanol and groundnut oil at the ratio of 1:1) to young adult male rats via a micropipette for 30 days and observed in the 10 mg/kg bw group statistically significant (*p* < 0.05) reductions in the cauda epididymal sperm count and sperm motility, statistically significantly reduced absolute weight of the testes and caput, corpus and cauda epididymides, and reduced activity of the antioxidant enzymes in the testes and corpus and cauda epididymides, accompanied by increased hydrogen peroxide generation and lipid peroxidation. In this study, the observed effects in the 10 mg/kg bw group on epididymal sperm count and testicular and epididymal weight were markedly smaller than in the study by Malini and co-workers. In the high-dose group (100 mg/kg bw), the effects were more pronounced and additionally included statistically significantly reduced sperm viability and significantly reduced relative testes weight (relative to body weight) (for details, see Table 1). Further immunofluorescence studies by the same group [61] revealed a dose-dependent increase in caspase 3 and FAS protein in testicular germ cells that was related to piperine administration and was accompanied by dose-dependent changes in the testicular antioxidant system (reduced activity of antioxidant enzymes, increases in hydrogen peroxide generation and in lipid peroxidation). In the low-dose group (1 mg/kg bw), no relevant reproductive toxicological effects were observed in either of the studies by D’Cruz et al. [60,61]. These studies used small animal groups with only four animals each, which reduces the scientific significance of the study findings. 

In young adult male rats that orally received 10 mg piperine/kg bw/day (suspended in normal saline containing 0.5% carboxymethyl cellulose) for 60 days, Chinta and co-workers observed a statistically significantly (*p* < 0.05) reduced epididymal sperm count, sperm motility and sperm viability compared to the control group (the latter receiving the vehicle only), accompanied by histopathological changes in the testes and epididymides; statistically significantly increased serum gonadotropins (FSH, LH), with a reduced intra-testicular testosterone concentration; and statistically significantly reduced activity of anti-oxidant enzymes or other enzymes in the testes and epididymides (for details, see Table 1) [16,62]. Data on reproductive organ weights were also recorded in this study, but in one of the two publications dealing with this study, inconsistencies were noticed between the testes weights and calculated indexes or a coefficient based on testes and body weights [62]. For this reason, no further detailed information on the weights of the reproductive organs is provided here. After a recovery period of 60 days without piperine administration, the observed changes were reversible. 

Animals receiving the piperine dose (10 mg/kg bw) every 4th day in the same study displayed some of the abovementioned adverse effects that were also observed with the daily piperine administration, e.g., reduced sperm viability and mobility, which were less pronounced but in some cases still statistically significant (for details, see Table 1). With piperine administration (10 mg/kg bw) every 7th day, the individual parameters that were mentioned in relation to the daily piperine administration showed changes in the same direction but the changes were considerably milder and no longer statistically significant (e.g., sperm count, testicular testosterone). Chinta and co-workers concluded from their data that piperine might be a good lead molecule for the development of a reversible oral male contraceptive [16,62]. Animals receiving piperine every day displayed decreased body weight of about 10% after 60 days of piperine administration, which was not statistically significant. All animal groups receiving piperine (n = 6 per study group) showed statistically significantly reduced absolute liver weights (approximately 35–39% reduction compared to the control group) at the end of the piperine administration period. The authors did not comment on those data. These reductions were still existent in the group that had received piperine every day (about −9%) and the group that had received piperine every 4th day (about −41%) after the recovery period, without reaching statistical significance (*p* < 0.05). Due to the administration of piperine suspended with carboxymethyl cellulose, it is assumed that piperine was given as a bolus once per day.

In a fourth study with male rats (juvenile animals, 35 days old at baseline; n = 6 per group) that involved administration of 0, 5 or 10 mg piperine/kg bw/day for 30 days by gavage, stimulation of pubertal Leydig cell development (increased Leydig cell number and promoted maturation) and an inhibited spermatogenesis were observed. Regarding the latter effect, the authors only cite histological findings of the testes and epididymides and indicated that already the dose of 5 mg piperine/kg bw/day reduced the epididymal sperm count, but no concrete figures on sperm counts were provided. This lack of data presentation reduces the scientific weight of the evidence provided in this study. Serum testosterone levels were elevated and the FSH levels were lowered in both piperine groups [63,64]. In this regard, the findings in juvenile rats differ from those observed in older rats [59,62]. 

Overall, the findings from these four studies [16,58,59,60,61,62,63,64] largely point in the same direction, with some differences of observed adverse reproductive effects between young adult and juvenile male rats. Concordantly, in young adult male rats, reproductive toxicological effects, i.e., disturbed spermatogenesis (and accompanying effects on testes, epididymides and accessory male reproductive organs of different nature and degrees) were observed with intakes of 10 mg/kg bw/day [16,58,59,60,61,62]. The less pronounced effects, which were only partly statistically significant, were observed in adult male rats already at 5 mg/kg bw/day. From these studies, a LOAEL of 5 mg piperine/kg bw/day [58,59] and a NOAEL of 1 mg/kg bw/day [60,61] can be identified for the endpoint male reproductive toxicity (disturbed spermatogenesis). However, the study of D’Cruz and co-workers used a wide spacing between the tested piperine doses (factor of 10) [60,61]. In one study, adverse paternal reproductive effects observed with repeated daily piperine (bolus) doses of 10 mg/kg bw were reversible after piperine discontinuation for several weeks [16,62]. Based on information of the four studies on piperine administration, it can be assumed that piperine administration in these studies was carried out via bolus administration. In three of these four studies, piperine administration was via gavage, micropipette or gastric catheter, suggesting bolus administration. The piperine dosage form of the fourth study, i.e., piperine in carboxymethyl cellulose, suggests bolus application as well.

It is noted that these studies are afflicted with certain limitations (statistical analysis of organ weights mainly comprising data on the absolute organ weights and the data on relative organ weights not being available in most cases; small animal group sizes in some studies; in the study of Chen et al., only histological findings were cited but no concrete data on sperm counts were provided; and reduced absolute liver weights not having been reported in other animal studies at this daily dose and contradictory information on the parameters related to testes weights in the study of Chinta and co-workers). However, taken as a whole, the aggregated study findings all point in the same direction and the paternal toxicological reproductive effects, i.e., disturbed spermatogenesis, are corroborated by findings at different levels, such as histopathology, sperm parameters, hormonal changes and changes at the level of enzyme activities, as well as changes in absolute organ weights. The limited scientific significance of absolute organ weights is acknowledged; however, a statistically significant change in the relative testes weights was seen at least in one study [58] with daily doses of 10 mg/kg bw. The observed differences in hormone levels between the studies of Malini et al. and Chinta et al. on the one hand compared to Chen et al. on the other hand may be related to the different life stages of the investigated male animals (juvenile versus young adult rats) [16,58,59,62,63,64]. 

The question of whether the mode of piperine administration in the study by D‘Cruz and co-workers (piperine dissolved in 10% DMSO, ethanol and groundnut oil) [60,61] or in the study by Chinta and co-workers (together with carboxymethyl cellulose) [16,62] could possibly affect the bioavailability of piperine, leading to increased adverse effects, remains elusive. Adequate data to compare the influence of these modes of administration with the influences of the currently available piperine-containing dietary supplements and the food additives or galenic technics used in their manufacturing on the bioavailability of piperine, are currently not available.

In contrast to the largely consistent findings from the four studies cited above, different results are available from the 90-day toxicity study with rats used by EFSA (2015) for the evaluation of piperine as a flavouring agent, and which has already been described in (2) in Section 4.2.2 [29,32]. In this study, intakes of 0, 5, 15 or 50 mg piperine/kg bw/day were administered via feed. In male animals of the 5 and 50 mg/kg bw-groups, statistically significantly reduced relative epididymis weights (relative to brain weight) were observed. EFSA attributed only limited toxicological relevance to these findings due to the small changes and the non-existent dose dependence, and these changes were without histopathological findings (see also (2) in Section 4.2.2 and Table 1). This study did not include any specific examinations of sperm parameters or LH and FSH blood levels, as these types of investigations are not common in 90-day toxicity studies. 

In its assessment, EFSA (2015) did not address the findings of Malini et al. [58,59] and D’Cruz and co-workers [60,61], which were available at that time (since a review of the available scientific literature was not foreseen at this time as part of this assessment procedure).

A major difference between the four animal studies cited first [16,58,59,60,61,62,63,64] and the 90-day toxicity study used by EFSA seems to be that in the 90-day toxicity study, piperine was administered via feed, resulting in multiple intakes of small quantities spread throughout the day, whereas in the four first-cited animals studies, it can be assumed that piperine was administered as a bolus dose, possibly resulting in higher maximum blood or tissue levels or otherwise increased bioavailability. The bolus administration of piperine in the first four animal studies more closely resembles the usual human use of food supplements, which often bear recommendations relating to 1–3 doses per day. 

In this context, it is noted that Daware et al. observed no increased numbers of abnormal sperm cells in a sperm shape abnormality test performed with male mice receiving daily doses of 35–75 mg piperine/kg bw for 5 days [54]. However, this test is primarily performed regarding a genotoxicity assessment.

In in vitro studies, reduced viability and motility of goat sperm cells were seen with high doses of piperine (40–100 µmol/L) added to the sperm culture media [65], as well as impaired fertilization ability of hamster sperms directly exposed to high piperine doses (180–1005 µmol/L) in the capacitation medium [66]. However, the scientific relevance of these in vitro findings remains elusive due to the high piperine concentrations used and the direct exposure of the sperm cells to piperine via culture media, which differs from the exposure of sperm cells resulting from oral piperine intakes. 

The mode of action of the bolus doses of piperine on spermatogenesis and the accompanying effects on male reproductive organs remains elusive. With young adult male rats, it has been hypothesized that induced oxidative stress due to depletion of antioxidant enzymes and increased generation of reactive oxygen species (ROS) in epididymis and testis, and activation of the Fas-mediated pathway in testicular germ cells, may contribute to the observed antifertility effects. However, inhibition of the cytochrome P-450 enzymes or other enzymes involved in the synthesis of testicular steroid hormones, interaction of piperine with the active site of the androgen binding protein, induction of hormonal imbalances (effects on serum levels of FSH, LH, sex hormone-binding globulin and testicular testosterone) or other effects on the functional integrity of the testis and the male reproductive organs are also being discussed, and appear possible [16,61,62].

It is noted that even with high bolus doses of fine *Piper nigrum* fruit powder (25 or 100 mg/kg bw/day) administered for 20 or 90 days to male mice, negative effects on the sperm count in the cauda epididymis, sperm motility, viability and number of morphologically abnormal spermatozoa were observed (viability not affected with 25 mg/kg bw dose administered for 20 days), which increased with escalating daily dose and duration of application from 20 to 90 days. After 90 days of pepper powder administration, statistically significantly reduced relative weights (relative to body weight) of the testis, epididymis and seminal vesicle were observed in both dosage groups. No male animal receiving 100 mg/kg bw/day for 90 days (other animals were not examined) was fertile in mating trials with untreated female mice 24 h and 14 days after the termination of pepper administration, respectively. The fertility of the treated male animals improved after an 8-week recovery period, but was still (statistically not significantly) reduced at this time point [67]. The fine fruit powder (suspended in water containing milk powder) was administered by a feeding needle; therefore, it can be assumed that the administration occurred as a bolus.

(4) Maternal Reproductive Toxicological and Embryotoxic Effects

Depending on the time point of piperine administration before or during pregnancy, different reproductive toxicological or embryotoxic effects were observed in female animals.

In the study by Daware et al. (2000), young female mice (n = 6 per group) receiving 0, 10 or 20 mg piperine/kg bw/day for 14 days until the day of mating with untreated male animals displayed a statistically significantly reduced mating rate in the high-dose group (mating performance: 50% versus 83% in control group) and in both piperine dose groups a statistically significantly reduced fertility index (fewer mated animals became pregnant; fertility index: 60% and 66%, respectively). With this piperine administration protocol, the litter size of the pregnant animals and the growth of the pups were not affected. When the piperine doses (10 or 20 mg/kg bw/day) were administered to female mice from Day 1 through to Day 5 of gestation, significantly reduced implantation rates were observed in both dose groups, with implantations in 1 out of 6 mated animals each in the low- or high-dose group, respectively, versus 6 out of 6 mated animals in the control group. The post-implantation survival was not affected. In this study, piperine was given suspended in a formulation containing 1% carboyxmethyl cellulose, which was most likely done by bolus administration [54]. 

Significant implantation-inhibiting effects were also observed in another study by Piyachaturawat et al. (1982) with mice receiving oral bolus doses of 2 × 12.5 or 2 × 50 mg piperine/kg bw/day (dissolved in equal volume of DMSO and 95% ethanol; n = 19–21) from Day 2 through to Day 5 of gestation, with the implantation rates reduced by 71% and 90%, respectively, compared to the control group receiving the vehicle only (other oral doses not investigated). In addition, significant abortive effects were observed in mice given the same piperine bolus doses (2 × 12.5 or 2 × 50 mg/kg bw/day; n = 17–21) from Day 8 through to Day 12 of gestation (interrupted pregnancies in 58.8% and 71.4% of pregnant animals, respectively; other oral doses not investigated). Bolus doses of 25 mg piperine/kg bw/day given from gestation Day 15 onwards (n = 8) resulted in delayed labour and significantly increased number of dead foetuses (6.1 dead foetuses/litter vs. 0.3 dead foetuses/litter in the control group; other oral doses not investigated) [68].

In a study with female rats (n = 6), a reduced implantation rate (33% reduction) was observed with an oral piperine dose of 100 mg piperine/kg bw/day administered from gestation Day 1 through to Day 7 (other doses not investigated) [69].

In female hamsters, which received intra-gastric daily doses of 50 or 100 mg piperine/kg bw/day from Day 1 through to Day 4 of the oestrus cycle, followed by hormonally induced superovulation and artificial insemination with spermatozoa of untreated male animals, increased fertilization of eggs in the early phase of fertilization 9 or 24 h after insemination were observed compared to the control animals [70]. However, due to the applied methodology (hormonally induced superovulation which might interfere with piperine effects on female reproduction, artificial insemination, no information on pregnancy outcome), the scientific significance of these findings remains elusive regarding the effects of piperine on maternal reproduction or embryonic development.

In conclusion, from these studies, a LOAEL of 10 mg/kg bw/day can be identified with regard to adverse maternal reproductive and embryotoxic effects in mice [54]. This was the lowest daily dose investigated in these studies.

(5) Interactions with Drugs

Animal studies on the interactions of piperine with drugs are discussed together with the corresponding human studies in Section 4.2.4.

#### 4.2.3. Human Studies

(1) Intervention Studies

In human single-dose studies, piperine doses of 50 or 500 mg were applied either alone (50 mg piperine) or in combination (500 mg piperine) with curcumin. However, neither of both studies was designed to address piperine safety issues or provided data on the safety parameters (occurrence/absence of adverse events, haematological or clinical chemistry lab parameters) [45,71].

Human studies with repeated piperine administrations comprise only a small number of studies using piperine without concomitant administration of other substances (drug interaction studies with piperine-only run-in phases of 3–10 days and piperine doses of 15–20 mg/day) [72,73,74,75,76,77,78,79]. In addition, there are a number of other studies in which piperine (in several cases in form of highly piperine-enriched pepper extracts) was given in combination with other substances, e.g., curcumin, resveratrol, *Camellia sinensis* extract, herbal extracts or others, in order to increase the bioavailability or effectiveness of these substances [80,81,82,83,84,85,86,87,88,89,90,91,92,93,94,95,96,97,98,99,100,101,102,103,104,105,106,107,108,109,110,111,112,113,114,115,116]. Some of these piperine combination studies included control groups receiving piperine without concomitant administration of the other substances mentioned above, but these studies lacked control groups receiving no piperine, and were conducted in individuals suffering from different diseases and using various drugs during the course of the study, or no information on product tolerance was provided [88,89,99,116]. The combination studies frequently used piperine doses in the range of 4–15 mg/day with study durations of 4–17 weeks, but also other studies are available, in which 60 mg piperine/day administered for 4 days [90], doses of 10 mg/day for 6 months [96,97] or doses of 40 mg/day for 3 or 6 month, respectively [86,111], were used. However, available human studies with piperine administration were primarily conducted to evaluate the efficacy of piperine or the efficacy of the accompanying substances, and in most cases safety issues were only marginally addressed or reported. Most of the studies provided no information or only inadequate information on the occurrence or absence of adverse events and/or no data of the relevant safety lab parameters (a situation that is often found with substances used as ingredients in food supplements [117]). In this regard, it should be noted that the fact that no information on the absence or occurrence of adverse events was provided in several studies cannot be taken as a proof that actually no adverse events occurred [117]. In some of the available studies in which piperine was administered concomitantly with other substances, it is stated that no serious adverse events or severe undesirable effects were reported, leaving open questions regarding the less severe effects. A few studies with combined administration of piperine (4–10 mg/day or unspecified doses) with other substances (iron preparation, resveratrol, curcumin, multi-ingredients or others) reported that no adverse events occurred [82,83,101,106,111,112,113,118]. Individual studies with combined administration of piperine with other substances (a multi-ingredient product, curcumin) conducted in patients with non-alcoholic fatty liver disease or COPD reported the occurrence of adverse events, comprising gastrointestinal adverse effects (abdominal discomfort or diarrhoea in two studies with 3/40 individuals or 4/45 individuals, respectively; none in placebo groups with n = 40 or 12, respectively) or rash (in one study with 1/45 individuals; none in placebo group with n = 12) [84,103]. 

None of the identified human studies included investigations regarding the potential effects of piperine on male reproductive capacity (i.e., sperm parameters).

Taken together, due to insufficient data on safety parameters, the lack of investigations into the effects of piperine on human spermatogenesis and/or the combined administration of piperine with other substances conducted in most studies, the available human studies involving piperine administration provide no adequate scientific basis for the assessment of the possible health risks of oral intake of isolated piperine used as single ingredient and ingested in bolus form.

(2) Studies on Reproductive Toxicological Effects

No published human intervention study could be identified, which included investigations into the effects of bolus intakes of isolated piperine on male reproductive organs, reproductive capacity or sperm parameters. The same applies regarding the reproductive effects in women (pregnant women or women who intend to become pregnant).

In an epidemiological study, a statistically significant inverse association was observed between plasma testosterone concentrations and, among others, plasma piperine levels in healthy middle-aged men (median: 50 years) [119]. However, as already mentioned by the study authors, a statistical association does not imply causality and therefore the scientific significance of these data from this particular study alone regarding male reproductive effects remains elusive (i.e., piperine plasma levels could just be a lifestyle marker).

#### 4.2.4. Interactions of Piperine with Medicinal Products and Other Substances

The pharmacokinetic interactions of piperine with various chemically and pharmacologically diverse drugs have been observed in both human and animal studies. In most instances, interactions of piperine with drugs resulted in a better bioavailability of the investigated drugs, exemplified by increases in the maximum plasma/serum concentrations (C_max_) and/or increased AUC values (AUC = area under the curve) for the respective drugs. These effects are in line with the purported “bio-enhancing” activity of piperine.

In human studies, oral administration of 20 mg piperine/day (~ 0,29 mg piperine/kg bw based on a body weight of 70 kg) for one or several days resulted in improved bioavailability (elevated serum/plasma concentrations and/or elevated AUC values) of the following drugs: propranolol (antihypertensive drug), theophylline (bronchodilatory drug), phenytoin, carbamazepine (antiepileptic drugs), nevirapine (HIV-1 reverse transcriptase inhibitor), chlorzoxazone (muscle relaxant), diclofenac (non-steroidal anti-inflammatory drug) and fexofenadine (antihistaminic drug). In the cases involving administration of 20 mg of piperine/day, the increases in the drug C_max_ and AUC values were approximately 1.07- to 2.2-fold (C_max_) and 1.09- to 2.7-fold (AUC values), depending on the drug and drug dosage. Regarding rifampicin (antibiotic drug), such interactions were reported in the context of concomitant administration of 50 mg piperine/day (for details, see Table 2) [72,73,74,75,76,77,79,120,121,122]. 

For midazolam (sedative), prolonged and increased pharmacological effects of the drug (prolonged duration of sedation, increased number of individuals with amnesia) were observed with piperine administration of 15 mg/day for 3 days and subsequent midazolam administration on the 4th day [78]. 

For the substances β-carotene and coenzyme Q_10_, increased bioavailability was already observed in the case of combined administration of 5 mg piperine/day with 15 mg β-carotene/day for 14 days or with 120 mg coenzyme Q_10_/day, respectively, for 21 days [123,124]. Regarding curcumin, the C_max_ or AUC values were approx. 30 or 20 times higher, respectively, when administered together with 20 mg piperine/day [125].

In these human studies referred to above, piperine was administered once daily as a bolus and the piperine and/or drug administration was performed for one or more days according to different administration schedules. The effects of piperine were influenced by the dose of the administered drug and/or the duration of the piperine or the drug administration and/or possibly by the investigated population group, i.e., healthy individuals or individuals suffering from certain diseases (for details, see Table 2 and below). With the exception of the studies on β-carotene and coenzyme Q_10_, currently no published human study could be identified with simultaneous longer-term piperine and drug/substance administration to achieve steady-state levels of piperine and the drug/substance. 

In animal studies (rodents or rabbits), higher piperine doses were generally used (2.1–30 mg/kg bw/day), as compared to the doses employed in human studies in which interactions were investigated. The interactions described for the animal studies in most cases also resulted in increased bioavailability (increased serum/plasma C_max_ and/or increased AUC values) of the investigated drugs. However, interactions in animal studies were observed with several additional drugs, such as ibuprofen, nimesulide, oxyphenylbutazone (non-steroidal anti-inflammatory drugs); amoxycillin, ampicillin, norfloxacin, marbofloxacin, metronidazole (antibiotic drugs); glimepiride, nateglinide (antidiabetic drugs); simvastatin, rosuvastatin (lipid-lowering drugs); verapamil, diltiazeme (calcium channel blockers); sodium valproate (antiepileptic drug); darunavir ethanolate (HIV protease inhibitor); losartan (angiotensin II receptor type 1 antagonist); domperidone (antiemetic drug); almotriptan (anti-migraine drug); and fexofenadine (antihistaminic drug) [126,127,128,129,130,131,132,133,134,135,136,137,138,139,140,141,142,143,144]. 

Increased bioavailability in animal studies has also been observed for a number of other substances (for example for puerarin, resveratrol, emodin, linarin or cannabidiol).

In the case of the drugs nimesulide, oxyphenylbutazone, ibuprofen or nateglinide, the piperine-mediated elevated bioavailability was reported to also be associated with increased drug efficacy. Moreover, increased pharmacological effects, without concomitant measurement of drug levels, have been observed for pentobarbitone (short-acting barbiturate), pentazocine (opioid), sertraline (selective serotonin reuptake inhibitor), midazolam or diazepam [126,134,140,141,145,146]. 

By contrast, animal studies were also identified in which no improved drug bioavailability or a decreased bioavailability was observed for the combined administration of piperine with drugs, i.e., with cefadroxil, carbamazepine, warfarin or diltiazem [127,147,148,149]. 

For diltiazem, a reduced drug bioavailability was observed with administration of 10 or 20 mg of piperine/kg bw/day for 14 days and diltiazem administration on Day 15 (i.e., one day after termination of piperine administration). The repeated dosing of piperine in this study led to induced gene expression of the multidrug transporter P-glycoprotein, which may have played a role in limiting the bioavailability of diltiazem [147]. 

As a further example, co-administration of warfarin (2 mg/kg bw) with piperine (10 mg/kg bw) to rats resulted in a decrease in the warfarin C_max_ and AUC values by 32 and 20%, respectively, combined with a reduced clinical anti-coagulant effect of warfarin [149].

The influence of the study conditions (investigated species, investigated human population, duration of drug treatment and/or schedule of piperine application and piperine dose) on the results of available interaction studies is illustrated by data obtained with carbamazepine. In healthy humans receiving a single dose of carbamazepine (200 mg) on Day 11 after 10 days of piperine administration (20 mg/day), significant increases in C_max_ (1.7 fold) and AUC (1.5 fold) values were observed compared to the control group receiving carbamazepine only, which were attributed to piperine-mediated inhibition of the CYP3A4 enzyme [73]. In epilepsy patients, with long-time carbamazepine monotherapy and steady-state carbamazepine plasma levels, small increases in C_max_ (1.1 fold) and AUC (1.1) values were observed after administration of a single dose of 20 mg piperine combined with 500 mg carbamazepine [121]. In an animal study with rats receiving a single combined dose of carbamazepine with 3.5 or 35 mg piperine/kg bw, no significant changes in plasma levels (C_max_ or AUC-values) of carbamazepine and the major carbamazepine metabolite, carbamazepine-10,11-epoxide, were seen compared to the control group receiving carbamazepine only. Rats concomitantly receiving the same carbamazepine and piperine doses for 14 days showed significantly decreased plasma levels of carbamazepine (plasma C_max_ 25–39% reduced; plasma AUC 29–37% reduced). The plasma levels of carbamazepine-10,11-epoxide and brain levels of carbamazepine were also reduced in both piperine-treated groups, but reached statistical significance only in the high-dose group. Carbamazepine-10,11-epoxide brain levels were also reduced but reached no statistical significance. A decreased plasma concentration of carbamazepine was observed in the high-dose piperine group, despite decreased CYP3A2 protein expression in rat liver. The reduced carbamazepine drug and metabolite levels were mainly attributed to a reduced carbamazepine absorption (animals displayed increased defecation and wet faeces with piperine) and a decreased brain penetration of carbamazepine caused by piperine [148]. 

In addition to the abovementioned study conditions, it can be assumed that the concomitant administration of other bioactive substances together with piperine might also influence the effects of piperine on the bioavailability of the investigated drugs.

Concerning the improved bioavailability seen with several drugs, different underlying mechanisms have been discussed, such as improved drug absorption and/or inhibition of degradation or elimination. In this context, depending on the drug or investigated substance, different mechanisms for influencing absorption and different molecular targets relating to the metabolism of xenobiotics are at the center of discussion, viz. the unspecific gastrointestinal effects (increased splanchnic blood flow), altered membrane dynamics, inhibition of cytochrome P-450 enzymes (CYP3A4, CYP2E1, CYP2C9, etc.), inhibition of multispecific efflux transporters, such as P-glycoprotein, or other mechanisms [1,15,73,74,75,76,77,78,120,124,150]. 

It is noted that for the reduced bioavailability of diltiazem observed in an animal study, induction of P-glycoprotein has been considered as an underlying principle [147].

In summary, single or short-term administration of piperine bolus doses combined with several chemically and pharmacologically diverse drugs resulted in interactions, which in most cases led to increased drug bioavailability. However, it can be assumed that such interactions with piperine may also occur with other drugs that have not be tested in this respect so far. Such interactions may vary over time and depend on the used drug, drug dosage and piperine dosage, and may also possibly vary depending on concomitant administration of further bioactive substances. Appropriate investigations in humans are required with the drugs in question to further clarify this issue. Interactions (increased bioavailability of drugs) in humans were observed with several drugs at bolus doses of 20 mg piperine/day. For one drug (midazolam), increased clinical efficacy was reported with bolus administration of 15 mg piperine/day. The findings with β-carotene and coenzyme Q_10_ indicate that already bolus doses of 5 mg piperine/day may possibly cause interactions with certain substances or drugs.

## 5. Discussion

This review focuses on the evaluation of the safety of isolated piperine used as a single ingredient in food supplements, i.e., in bolus form, in adult individuals. Children and adolescents were not included in the consideration since adult persons constitute the prime target population for food supplements containing piperine. 

Piperine is a natural ingredient of *Piper nigrum* (black pepper) and some other *Piper* species, e.g., *Piper longum*. Due to the use of peppercorns as a spice, it is a common component of the human diet. Apart from this source, piperine may be used in isolated form as a flavouring agent in food production. 

When pepper is used for food seasoning purposes, piperine is added to food in combination with all other components of the peppercorns (which may influence observed biological effects) and in varying degrees of comminution (which may influence its bioavailability). In this context, it is usually consumed together with large quantities of food and in several portions throughout the day. By contrast, when piperine is used in food supplements, the piperine supply differs greatly in that piperine is ingested in isolated or highly concentrated form and as a bolus (usually in 1–3 portions per day), without any substantial amounts of other pepper components. These differences may influence the bioavailability and biological effects of piperine ingested as a bolus and in isolated form compared to its usual intake via food seasoning.

Currently available human studies with oral piperine administrations provide no adequate scientific basis for the final assessment of the potential health risks associated with intakes of isolated piperine used as a single ingredient and ingested in bolus form. This is a situation also frequently found with other substances used as ingredients of dietary supplements. Although hardly any adverse effects (apart from potentially undesirable interactions of piperine with various drugs) were reported in available human studies, it is noted that in most cases safety issues were only marginally addressed or reported. In addition, is to be considered that in many studies piperine was administered in combination with other substances and that the combined administration complicates data interpretation. 

However, some currently available human and animal studies already provided indications of potential health risks of bolus doses of isolated piperine.

In human and animal studies with single or short-time application, piperine was shown to interact with various drugs. In most cases, these interactions led to an improved bioavailability of the investigated drugs and are in line with the purported “bio-enhancing” activity of piperine. It can be assumed that such interactions may also occur with other drugs, and can vary, depending on the individual drug, drug dosage, piperine dosage, duration of intake and time span between piperine and drug intake. Additionally, interactions may also possibly vary depending on the concomitant administration of further bioactive substances. For clarification, appropriate investigations in humans are required with the drugs in questions, involving in particular studies with repeated drug and piperine application to reach steady-state levels. 

Improved bioavailability can offer advantages in drug therapy if this is performed under medical supervision. Without adequate medical supervision, however, depending on the drug, piperine-based drug interactions may carry the risk of unintended and/or deleteriously increased medicinal drug effects or of the occurrence of adverse drug effects, especially in the case of drugs with a narrow therapeutic range. Increased bioavailability of certain drugs has been observed in several human studies with bolus doses of 20 mg/day (= LOEL), and findings with β-carotene and coenzyme Q_10_ suggest that in some cases bolus doses of 5 mg piperine/day might also cause such interactions. Taken together, against the background of these data, it seems advisable that individuals taking medicinal products, especially drugs with known piperine interactions or drugs for which no interaction data are available, should consult a physician prior to the use of isolated piperine as a food supplement.

Based on animal data, further potential health risks or potential risk groups, respectively, of bolus doses of isolated piperine can be identified. In four animal studies with juvenile and young adult rats, largely consistent paternal reproductive toxic effects (see Table 1) were observed at piperine bolus doses of 10 mg/kg bw/day [16,58,59,60,61,62,63,64]. 

In young adult rats, male reproductive toxic effects, which were significantly weaker and only partly statistically significant, were observed by Malini et al., already at bolus doses of 5 mg/kg bw/day [58,59]. On the other hand, a NOAEL of 1 mg piperin/kg bw/day (as bolus) can be identified from the study by D’Cruz and co-workers, who used a large spacing between the tested piperine doses (factor 10) [60,61]. Consistently clearer adverse paternal reproductive effects, i.e., disturbed spermatogenesis and accompanying adverse male reproductive effects, however, were observed with doses of 10 mg/kg bw/day. 

It is to be acknowledged that these studies are afflicted with certain limitations; however, taken as a whole, the aggregated study findings point in the same direction and paternal toxicological reproductive effects, i.e., disturbed spermatogenesis, are corroborated by findings at different levels (i.e., histopathology, sperm parameters, hormonal changes and other parameters).

The reason for the different findings in terms of paternal reproductive toxic effects of these four studies compared to a sub-chronic 90-day animal toxicity study [29] remains elusive. The major difference seems to lie in the method of piperine administration: in the sub-chronic 90-day animal study, piperine was administered via feed, resulting in multiple intakes of small piperine quantities spread throughout the day. In the case of the other four abovementioned animal studies, piperine administration as a bolus can be assumed, possibly resulting in higher peak blood or tissue levels or otherwise increased bioavailability. 

Regarding the reversibility of the disturbed spermatogenesis observed by Chinta and colleagues (see above) [16,62], it remains to be investigated to what extent such reversibility may still be present with piperine administrations lasting longer than 60 days. In addition, it remains to be clarified to what extent these findings may be extrapolated to humans. Even taking into account these findings of Chinta and co-workers with respect to the potential reversibility of the perturbation of spermatogenesis under certain conditions, the consistent paternal reproductive effects observed with bolus doses of 10 mg/kg bw/day should be classified as being toxicologically relevant and of potential concern. 

Human studies that included adequate investigations to clarify the possible effects of bolus doses of isolated piperine on the male reproductive system could not be identified in the course of preparation of the current review. Therefore, as long as this knowledge gap has not been resolved and against the background of the adverse male reproductive effects observed in animal studies with high bolus intakes of isolated piperine, it seems advisable to maintain an adequate margin of exposure between those bolus doses of isolated piperine for which adverse male reproductive effects in animal studies were reported and the daily amounts of isolated piperine used in food supplements. 

By using a dose of 10 mg piperine/kg bw/day for which adverse male reproductive effects were reported in several animal studies as a point of departure, the application of an uncertainty factor of 3 seems warranted for extrapolation to a NOAEL from this intake level. In addition, for deriving health-based guidance values from animal data, EFSA recommends to use an overall default assessment factor of 100 to account for inter-species and intra-human variability (10 for inter-species variability x 10 for inter-human variability) in the absence of chemical-specific data on the kinetics and/or dynamics [151]. Based on an assumed body weight of 70 kg, this approach would lead to a health-based guidance value of 2.3 mg/day (10 mg/kg bw/day × 70 kg bw/(3 × 100)) for piperine when used in isolated form via bolus administration, i.e., as a food supplement. This intake seems to be low compared to estimates of daily piperine intakes resulting from the usual culinary use of pepper in normal human diet. However, it should be taken into account that the intake of piperine in isolated form may not be directly comparable to its intake in conjunction with all other pepper ingredients and various degrees of comminution of the peppercorn. On the other hand, the effect of pepper consumption (in particular of high bolus doses with a high degree of comminution) on human male reproductive capacity remains to be clarified. 

The health-based guidance value of 2.3 mg/day, as calculated above, which would currently be expected to provide an adequate level of protection with respect to potential male reproductive toxicity, would also be below the piperine bolus doses for which interactions with concomitantly administered drugs/substances have been observed.

In animal studies, maternal reproductive toxicity and embryotoxic effects were observed with piperine bolus doses of 10–25 mg/kg bw/day, which varied, depending on the time of piperine administration with regard to the day of mating, the duration of time covered during gestation and the piperine dose (10 mg/kg bw/day: reduced fertility index, reduced implantation rates; 25 mg kg bw/day: reduced implantation rates, abortive effects, delayed labour and increased foetal mortality) [54,68]. It should be noted that the observed adverse effects occurred in these studies at the lowest piperine bolus doses tested and that certain adverse effects were only investigated at 25 mg/kg bw/day. From the available animal studies, a LOAEL of 10 mg/kg bw/day, but no NOAEL regarding maternal reproductive toxicity and embryotoxic effects could be identified. It remains to be clarified whether the use of DMSO in the oral piperine application by Piyachaturawat et al. (1982) might have had an influence on the bioavailability or observed effects of piperine. However, adverse maternal reproductive and embryotoxic effects were also observed in a second study [54]. 

With regard to the potential health risks for which there are indications when using isolated piperine in bolus doses (drug interactions, disturbed spermatogenesis, adverse maternal reproductive and embryotoxic effects), it remains to be clarified whether these risks may be influenced by other bio-active, additional ingredients in food supplements ingested together with piperine. In principal, the same considerations apply to highly piperine-enriched pepper extracts, although the difference between some highly piperine-enriched extracts and the piperine preparations supplied from chemical companies (purity: ≥ 97%), used for instance in the cited studies of Chinta et al. [16,62] or D’Cruz et al. [60,61], does not seem to be substantial. However, in cases involving either other bio-active ingredients and/or highly piperine-enriched pepper extracts, any claimed mitigation or elimination of the adverse effects described above should be supported by adequate scientific investigations and data.

In conclusion, human and animal studies with single or short-term application of isolated piperine used in bolus form revealed interactions of the substance with several drugs, which can give rise to potential health risks, and based on which individuals taking medications can be identified as a potential risk group. For individuals taking medicinal products (especially drugs with known piperine interactions or drugs for which no interaction data are available), it seems advisable to consult a physician prior to the use of isolated piperine as a food supplement.

Animal studies with higher daily piperine bolus doses provide indications for further potential health risks (disturbed spermatogenesis; adverse maternal reproductive and embryotoxic effects), for which no adequate human data are currently available. 

Considering that a distinct NOAEL for maternal reproductive and embryotoxic effects could currently not be identified from the available animal studies, it seems advisable for pregnant women to abstain from the use of food supplements containing isolated piperine. Moreover, the reduced implantation rates that were reported in animal studies may also be of relevance for women who wish to become pregnant.

Regarding the observed adverse paternal reproduction effects in animal studies and the lack of information on the effects of bolus doses of isolated piperine on the human male reproductive system, it appears prudent to maintain an adequate margin of exposure between those bolus doses that produced adverse paternal reproductive effects in animal studies and the maximum daily amounts of isolated piperine in food supplements. 

Considering the uncertainties outlined above, the importance of addressing the existing knowledge gaps regarding effects of bolus doses of isolated piperine on human male reproductive capacity in future human intervention studies, by including specific investigations into this endpoint in the study design, is emphasised.

## Figures and Tables

**Table 1:** Paternal reproductive effects of piperine observed in animal studies.

Authors	Species	Application Mode	Duration	Dosage	Effects
(1) Studies for which bolus application of piperine can be assumed					
(1.1) Studies with male rats with the age of ≥90 days at study start (young adult rats)					
Malini et al., 1999 [58,59]	male albino rats (3–4 months old)	suspended in 0.9% saline administered via gastric catheter	30 days	5 mg/kg bw/day	histopathology: partial degeneration of germ cell types. serum LH stat. significantly ^1^ increased. absolute organ weight of cauda epididymides and vas deferens stat. significantly reduced. Effects on other parameters mentioned below with the administration of 10 mg piperine/kg bw/day (sperm concentration, relative weight of testes, absolute weight of seminal vesicle and ventral prostate, serum FSH and intratesticular testosterone level) were in the same direction as seen with the dose of 10 mg/kg bw/day, but the changes were milder in nature and did not reach statistical significance.
				10 mg/kg bw/day	sperm concentrations in caput and cauda epididymides stat. significantly reduced. histopathology: severe damage to seminiferous tubule, decreased seminiferous tubular and Leydig cell nuclear diameter, desquamation of spermatocytes and spermatids. serum FSH and LH stat. significantly increased; intratesticular testosterone stat. significantly decreased. relative organ weight of testes stat. significantly reduced; absolute organ weight of cauda epididymides, vas deferens, seminal vesicle and ventral prostate stat. significantly reduced. total lipids in testes stat. significantly reduced; changes in testicular lipid composition. enzyme activities in testes of NAD+-dependent malate dehydrogenase and NADP+-isocitrate dehydrogenase stat. significantly decreased.
D‘Cruz and Mathur, 2005; and D‘Cruz et al., 2008 [60,61]	male Wistar rats (90 days old)	dissolved in vehicle (10% DMSO in ethanol and groundnut oil ration of 1:1) administered via micropipette	30 day	1 mg/kg bw/day	no relevant effects on sperm parameters, weight of testes and accessory sex organs or investigated enzyme activities.
				10 mg/kg bw/day	stat. significantly decreased epididymal sperm count and sperm motility. stat. significantly reduced absolute organ weight of testes and caput, corpus and cauda epididymides. stat. significantly reduced levels of sialic acid in testes and caput epididymides. reduced activity of antioxidant enzymes in testes and corpus and cauda epididymides (in most cases statistically significant). stat. significantly increased hydrogen peroxide generation in testes and corpus and cauda epididymides.
				100 mg/kg bw and day	stat. significantly decreased epididymal sperm count, sperm motility and sperm viability. significantly reduced relative organ weight of testes (as indicated in text, data not shown); stat. significantly reduced absolute organ weight of caput, corpus, cauda epididymides, seminal vesicle and ventral prostate. stat. significantly reduced levels of sialic acid in testes and caput, corpus and cauda epididymides, stat. significantly reduced activity of antioxidant enzymes in testes and epididymides. stat. significantly increased hydrogen peroxide generation in testes and caput, corpus and cauda epididymides.
Chinta and Periyasamy 2016; and Chinta et al., 2017 [16,62]	male Wistar rats (90 days old)	suspended in 0.5% carboxymethyl cellulose (no further information on application mode)	60 days of piperine administration followed by recovery period of 60 days without piperine administration	10 mg/kg bw administered every day	Effects at the end of **the piperine administration period ^2^** stat. significantly decreased epididymal sperm count, sperm motility and sperm viability. histopathology: desquamated spermatozoa and decreased thickness of germ layer in seminiferous tubules; histopathological changes in epididymides and seminal vesicle. serum FSH, LH and sex hormone-binding globulin stat. significantly increased; testicular testosterone stat. significantly decreased. stat. significantly reduced level of sialic acid in epididymides and fructose level in seminal vesicles, stat. significantly reduced activity of antioxidant enzymes (super oxide dismutase, catalase) in testes and epididymides. stat. significantly increased lipid peroxidation in testes and epidydimides. in Leydig cells stat significantly decreased activity of enzymes involved in testosterone synthesis absolute liver weight was reduced by approximately 36% (stat. significant) **After a recovery period** of 60 days without piperine administration, the observed adverse effects on reproductive organs were reversible Absolute liver weight was still reduced by approximately 29% (stat. not significant).
				10 mg/kg bw administered every 4th day	Effects at the end of **the piperine administration period** ^2^ With this administration regime, several of the adverse effects seen with the daily piperine administration were also observed but these changes were smaller, however, in some cases still statistically significant: (i.e., stat significantly reduced sperm motility and viability; stat significantly reduced testicular testosterone; stat significantly reduced levels of sialic acid in epididymides and fructose in seminal vesicle; stat significantly reduced activity of antioxidant enzymes in epididymides). Some other changes did not reach statistical significance. Absolute liver weight was reduced by approximately 35% (stat significant). **After a recovery period** of 60 days without piperine administration the observed adverse effects on reproductive organs were reversible with no significant deviations from control group. Absolute liver weight was still reduced
				10 mg/kg bw administered every 7th day	Effects at the end of the **piperine** **administration period** Sperm count or testicular testosterone were somewhat reduced but without reaching statistical significance. Absolute liver weight was reduced by approximately 39% (stat. significant). **After a recovery period** of 60 days without piperine administration, no significant deviations from control group
** (1.2) Studies with male rats with age of 35 days at study start (juvenile rats)**					
Chen et al., 2018 [63,64]	male Sprague-Dawley rats (35 days old)	suspended in normal saline administration via gavage	30 days	5 mg/kg bw/day	Dose-dependent increase in serum testosterone; decrease in serum FSH (similar serum levels in both piperine groups). increase in Leydig cell size and slight increase in Leydig cell number (similar increases in both piperine groups). authors state that sperm count in epididymides was reduced (only pictures of histological organ sections shown but no data on sperm count provided) [64].
				10 mg/kg bw/day	dose-dependent increase in serum testosterone; decrease in serum FSH (similar serum levels in both piperine groups). Increase in Leydig cell size and slight increase in Leydig cell number (similar increases in both piperine groups). authors state that sperm count in testis and epididymides was reduced (only pictures of histological organ sections shown but no data on sperm count provided).
**(2) Studies with piperine application spread over the course of the day**					
Bastaki et al., 2018 [29]	female and male Sprague-Dawley rats (48–57 days old)	added to feed	90 days	5 mg/kg bw/day	statistically significant decrease of relative (to-brain ratio) epididymides weight (by about 23% compared to control) without histologic correlates ^3^.
				15 mg/kg bw/day	statistically not significant decrease of relative (to-brain ratio) epididymides weight (by about 17% compared to control) without histologic correlates ^3^.
				50 mg/kg bw/day	statistically significant decrease of relative (to-brain ratio) epididymides weight (by about 21% compared to control) without histologic correlates ^3^.

^1^ stat. significantly = statistically significantly; *p* < 0.05; ^2^ Data on male reproductive organ weights were also recorded in this study, but in one of these two publications inconsistencies were noticed between the testes weights and calculated indexes or a coefficient based on testes and body weights [62]. For this reason, no further detailed information on the weights of the male reproductive organs is provided here. ^3^ Only potentially reprotoxicologically relevant effects reported. For other effects observed in this study, see (2) in Section 4.2.2.

**Table 2:** Effects of piperine on the bioavailability of drugs in human studies.

Drug	Therapeutic Use	Drug Dose and Duration	Piperine Dose and Duration	Drug Bioavailability	Reference
propranolol	antihypertensive drug	40 mg/day as single dose, preceded by piperine administration for 7 days	20 mg/days for 7 days	increased bioavailability (AUC: 2.03 fold, C_max_: 2.04 fold)	Bano et al., 1991 [72]
theophylline	bronchodilatory drug	150 mg as single dose, preceded by piperine administration for 7 days	20 mg/days for 7 days	increased bioavailability (AUC: 1.96 fold, C_max_ 1.62 fold)	
rifampicine	antibiotic drug	450 mg as single dose	50 mg as single dose	increased bioavailability (AUC: 1.71 fold, C_max_: 1.25 fold)	Zutshi et al., 1985 [122]
phenytoin	antiepileptic drug	300 mg as single dose preceded by piperine administration for 7 days, given to healthy subjects	20 mg/day for 7 days	increased bioavailability (AUC: 1.50 fold, C_max_: 1.27 fold) C_max_ increase was statistically not significant	Bano et al., 1987 [79]
		150 mg given to patients with epilepsy who were on phenytoin therapy with 2 × 150 mg/day for at least 2 months	20 mg as single dose	slightly increased bioavailability (AUC: 1.09 fold, C_max_: 1.10 fold)	Pattanaik et al., 2006 [120]
		200 mg given to patients with epilepsy who were on phenytoin therapy with 2 × 200 mg/days for at least 2 months	20 mg as single dose	slightly increased bioavailability (AUC: 1.17 fold, C_max_: 1.22 fold)	
carbamazepine	antiepileptic drug	200 mg as single dose given to healthy subjects on day 11 (applied 1 day after last piperine dose)	20 mg/day for 10 days	increased bioavailability (AUC_last_: 1.48 fold, C_max_: 1.68 fold)	Bedada et al., 2017 [73]
		300 mg given to patients with epilepsy who were on carbamazepine therapy with 2 × 300 mg/day for at least 2 months	20 mg as single dose	slightly increased bioavailability (AUC: 1.10 fold, C_max_: 1.07 fold; C_max_ increase was statistically not significant	Pattanaik et al., 2009 [121]
		500 mg given to patients with epilepsy who were on carbamazepine therapy with 2 × 500 mg/day for at least 2 months	20 mg as single dose	slightly increased bioavailability (AUC: 1.13 fold, C_max_: 1.10 fold)	
nevirapine	non-nucleoside HIV-1 reverse transcriptase inhibitor	200 mg as single dose on day 7 of piperine administration	20 mg/day for 7 days	increased bioavailability (AUC: 2.67 fold, C_max_: 2.20 fold)	Kasibhatta and Naidu 2007 [77]
chlorzoxazone	centrally acting muscle relaxant	250 mg as single dose on day 11 (applied 1 day after last piperine dose)	20 mg/day for 10 days	increased bioavailability (AUC_∞_: 1.69 fold, C_max_: 1.58 fold)	Bedada and Boga 2017 [74]
fexofenadine	antihistaminic drug	120 mg as single dose on day 11 (applied 1 day after last piperine dose)	20 mg/day for 10 days	increased bioavailability (AUC: 1.68 fold, C_max_: 1.88 fold)	Bedada and Boga 2017 [75]
diclofenac	nonsteroidal anti-inflammatory drug	100 mg as single dose on day 11 (applied 1 day after last piperine dose)	20 mg/day for 10 days	increased bioavailability (AUC: 1.66 fold, C_max_: 1.64 fold)	Bedada et al., 2017 [76]
midazolam	sedative	10 mg as single dose on day 4 (applied 1 day after last piperine dose)	15 mg/day for 3 days	increased clinical effects ‐ increased duration of sedation ≈ 1.89 fold; ‐ increased number of individuals with drug-induced amnesia	Rezaee et al., 2014 [78]

## Data Availability

Not applicable.

## Abbreviations

AUC	area under the curve
bw	body weight
C_max_	maximum (or peak) serum/plasma concentration of a drug/substance
CMEC	(Australian) Complementary Medicines Evaluation Committee
COPD	chronic obstructive pulmonary disease
DMSO	dimethyl sulfoxide
EFSA	European Food Safety Authority
FAO	Food and Agriculture Organization
FSH	follicle-stimulating hormone
i.g.	intragastric
i.v.	intravenous
JECFA	Joint FAO/WHO Expert Committee on Food Additives
LH	luteinizing hormone
LOAEL	Lowest observed adverse effect level
LOEL	Lowest observed effect level
MOE	Margin of Exposure
MSDI	Maximised Survey-derived Daily Intake
NOAEL	No observed adverse effect level
NOEL	No observed effect level
OECD	Organization for Economic Co-operation and Development
mTAMDIs	Modified Theoretical Added Maximum Daily Intakes
TGA	(Australien) Therapuetic Goods Administration
T_max_	time to reach maximum serum/plasma concentration after administration of a drug/substance
WHO	World Health Organization
